# Carbon Foam-Reinforced Polyimide-Based Carbon Aerogel Composites Prepared via Co-Carbonization as Insulation Material

**DOI:** 10.3390/gels8050308

**Published:** 2022-05-16

**Authors:** Zixuan Zheng, Guojie Liang, Li Li, Jing Liu, Xinbo Wang, Yi Sun, Kai Li

**Affiliations:** State Key Laboratory of NBC Protection for Civilian, Research Institute of Chemical Defense, 35 Huayuan North Road, Haidian District, Beijing 100083, China; zzx19972022@163.com (Z.Z.); lily97@buaa.edu.cn (L.L.); liuj200721296@sina.com (J.L.); wxb1993@mail.ustc.edu.cn (X.W.); 13501260901@139.com (Y.S.)

**Keywords:** polyimide aerogel, carbon aerogel, thermal conductivity, shrinkage, co-carbonization mechanism

## Abstract

The weak inherent non-covalent interactions between carbon aerogel backbone nanoparticles obtained by the pyrolysis of conventional organic aerogel can lead to poor mechanical properties. When applied in the thermal protection system of a high-speed spacecraft, the preparation of carbon aerogel insulation materials with excellent formability and high mechanical strength still remains a huge challenge. This work reports an efficient approach for fabricating carbon foam-reinforced carbon aerogel composites by compounding the nanoporous polyimide aerogel into the microporous pre-carbonized phenolic resin-based carbon foam via vacuum impregnation, gelatinizing and co-carbonization. Benefiting from the co-shrinkage caused by co−carbonization, the thermal insulation capacity of the carbon aerogel and the formability of the pre−carbonized foam are efficiently utilized. The shrinkage, density and carbon yield of aerogels, pre-carbonized foams and the composites at different temperatures were measured to analyze the formation of the interfacial gap within the composite. The co-carbonization mechanism of the polyimide aerogels and phenolic resin-based pre-carbonized foams was analyzed through XPS, TG-MS, and FT-IR. Among the prepared samples, CF30-CPI-1000 °C with small interfacial gaps showed the lowest thermal conductivity, which was as low as 0.56 W/(m·K) at 1900 °C, and the corresponding compressive strength and elastic modulus were as high as 0.532 MPa and 9.091 MPa, respectively.

## 1. Introduction

Given the current thermal protection issues of high-speed spacecrafts resulting from consistent breakthroughs of the technical barrier of flight speed, the discovery of new thermal insulation materials has become increasingly imperative in order to support long-life and long-duration flight. Ideal thermal insulation materials for high-performance space vehicles should be lightweight, robust and thermally stable [1,2]. In particular, the aerogel and aerogel-derived materials have aroused enormous attention considering their nanoscale pore structure, extremely low density (up to 90% porosity) and low thermal conductivity. Therefore, aerogel materials have been recognized as the candidate for insulation material as they can effectively inhibit solid-phase heat transfer, airflow convective heat transfer, and radiation heat transfer [3].

So far, many types of aerogels have been investigated, including oxide, sulfide, carbide, metal, organic and carbon aerogels [4]. Carbon aerogels consisting of nano-skeletons combine the advantages of carbon-based materials and aerogel materials. Due to their low expansion coefficients, carbon aerogels maintain the nanoporous thermal insulation structure and the highest thermal stability under non-oxidizing atmospheres over 2800 °C. In comparison with the specific extinction coefficient of SiO_2_ aerogel after doping with shading agent (50 m^2^/kg), [5] the high specific extinction coefficient carbon skeleton of carbon aerogel over 1000 m^2^/kg [6] has strong shielding effects on the infrared radiation thermal conduction at high temperature [3]. Therefore, carbon aerogels with thermal conductivity as low as 0.01 W/(mK) at 1500 °C [7] are a type of ultra-high temperature thermal insulation material that has great potential application in thermal protection systems of hypersonic space vehicles that are subjected to ultra-high temperature and high heat flux.

In 1981, Pekala et al. [8] prepared the first organic aerogel from resorcinol–formaldehyde. Since then, the carbonization of organic aerogels under high temperature in an inert atmosphere has become an usual way to prepare carbon aerogel. A variety of organic aerogels including cresol–formaldehyde [9], melamine–formaldehyde [4], phenolic resin–furfural [10], polyimide [11] and other organic polymer aerogels [7] have been reported as the carbonization precursors. In particular, polyimide aerogels, comprised of a unique imide ring (–CO–N–CO–) structure, have inspired great interest over the past decades [12]. This characteristic of molecular structure contributes to the ultra-high specific surface area and microporosity, resulting in excellent heat resistance of the polyimide aerogels [12]. Therefore, carbon aerogels derived from polyimide have a great potential not only in the applications of photocatalysis, electrochemistry, and adsorption [11,13,14] but also in high-temperature thermal protection.

However, the carbonization of polyimide aerogels can lead to undesirable shrinkage and curling, leading to poor mechanical strength and irregular morphology of aerogel, which limits their practical applications as thermal insulation structural materials [15]. To address this issue, a series of reinforced aerogels were developed using fibers [16,17,18], graphene oxide [19,20], graphene [21,22], montmorillonite [23], and cellulose [14] as reinforcements. Because carbon foams with a three-dimensional microporous structure formed by the template method have low density, low thermal conductivity, and regular continuous phase morphology [24], they can be adopted to reinforce carbon aerogels to improve the processability and designability of the latter and simultaneously improve the thermal insulation and mechanical properties of the composites.

Carbon foam-reinforced carbon aerogel composites can be prepared by impregnating the aerogel precursor solution into the carbon foam under vacuum conditions, followed by gelation, drying, and carbonization. The difference of shrinkage factor between impregnated aerogel and carbon foam skeleton during supercritical drying and carbonization can lead to gaps at the interface of these two phases, which will increase the thermal conductivity of the composites and eventually deteriorate the thermal resistance of the materials [16]. Therefore, it is crucial to reduce the shrinkage discrepancy between aerogel and carbon foam to downsize the interfacial gap of the two phases.

In this work, biphenyl-type diamine and dianhydride monomers with strong molecular anisotropy were selected as raw materials to prepare polyimide aerogels and then carbonized to obtain polyimide-based carbon aerogels. On the other hand, the pre-carbonized foam (pyrolyzed at 500 °C) was used as reinforcement. After supercritical drying, the aerogel and pre-carbonized foam synchronously shrink during co-carbonizing up to 1000 °C to reduce the gaps between the aerogel and the foam struts. In addition, the co-carbonization mechanisms of the aerogel and carbon foam were carefully discussed. The prepared carbon foam-reinforced carbon aerogel composites showed low thermal conductivity and high mechanical strength. The influences of carbonization temperature and the foam density on the structure and properties of the composites were systematically investigated.

## 2. Materials and Methods

### 2.1. Materials

Polyurethane foam was obtained from Langfang Zhuosheng Building Materials Co., Ltd. (Langfang, China), with an apparent density of 0.035 g/cm^3^. Borated phenolic resin was obtained from Anhui Tianyu Plastic Co., Ltd. (Bengbu, China) with a density of 1.30 g/cm^3^. 1,3,5-Triaminophenoxybenzene (TAB) was obtained from Shanghai Yuanye Biotechnology Co., Ltd. (Shanghai, China). Pyridine, acetic anhydride, acetone, ethanol and anhydrous N-methylpyrrolidinone (NMP) were purchased from Sinopharm Chemical Reagent Beijing Co., Ltd. (Beijing, China). 2,2′-Dimethylbenzidine (DMBZ) and biphenyl-3,3′,4,4′-tetracarboxylic dianydride (BPDA) were obtained from Shanghai Aladdin Industrial Co., Ltd. (Shanghai, China). Dianhydride was dried in vacuum at 125 °C for 24 h before use. All other reagents were used without further purification.

### 2.2. Preparation of Pre-Carbonized Foam

The phenolic resin-ethanol solution with mass fraction of 20%, 30%, 40%, 50% was prepared at 60 °C. The polyurethane foam as template was fully impregnated in the above solution and then dried in a blast oven at 90 °C to remove the residual ethanol. The temperature was further raised to 165 °C at 0.5 °C/min and kept constant for 3 h to allow the crosslinking and curing reaction between molecular chains, aiming to obtain the foam stabilized by oxidation. Finally, the precursor foam was heated at a rate of 2 °C/min in a carbonizing furnace (model: KMTF-1200-I-60-340) under nitrogen atmosphere and pre-carbonized at 500 °C for 3 h. The pre−carbonized foam reinforcements are denoted as CFx-500 °C, where x represented the mass fraction of phenolic resin-ethanol solution.

### 2.3. Preparation of Sol–Polyimide Precursor Solution

Polyimide aerogels were prepared by controlling the molar ratio of dihydride (BPDA) and diamine (DMBZ). Firstly, DMBZ (10.08 g, 47.4 mmol) was dissolved with 150 mL NMP solution at nitrogen atmosphere under continuous agitation. BPDA (14.34 g, 48.9 mmol) pre-dried under vacuum at 125 °C for 24 h was added to the DMBZ solution at 20 °C. Then, 30 mL of NMP solution dissolved with TAB (0.42 g, 1.05 mmol) was added to the above solution. After stirring for 15 min, the dehydrating agent acetic anhydride (36.90 mL, 390 mmol) and catalyst pyridine (31.50 mL, 380 mmol) were added to obtain transparent and viscous sol-polyimide precursor solution.

### 2.4. Preparation of Carbon Aerogel and Carbon Foam-Reinforced Carbon Aerogels

**Figure 1:**
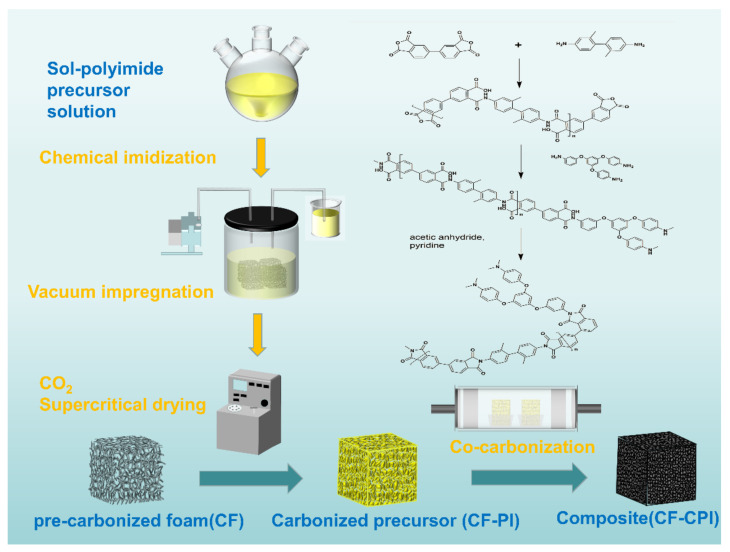
Preparation diagram of polyimide-based carbon aerogel-reinforced carbon foam composites.

The sol–polyimide was rapidly injected into CFx under vacuum condition and gelled within 15 min. The impregnated composite gel and the remaining wet gel were aged at 25 °C for 24 h and then immersed into 100% acetone. The solvent was exchanged for three times at 24 h intervals and then removed by CO_2_ supercritical drying. Polyimide aerogels (PI) and the composite precursor (CFx-PI) were obtained by vacuum drying overnight at 80 °C. Under the nitrogen protection, PI and CFx-PI were carbonized in a horizontal tubular furnace. The samples were calcined by increasing the temperature to 350 °C at a rate of 2 °C/min and kept constant for 1 h. Then, the carbonization was continued to the targeting temperature of 800 °C, 900 °C and 1000 °C with a rate of 1 °C/min and maintained for additional 1 h. Carbon foams and carbon aerogels were denoted by CFx-y and CPI-y, and the composite samples were denoted by CFx-CPI-y, where x represents the mass fraction of phenolic resin ethanol solution and y represents the pyrolysis temperature. The preparation diagram of composites is shown in Figure 1.

### 2.5. Characterization

The morphologies of the samples were characterized by scanning electron microscope (SEM, ZEISS Gemini 300). The N_2_ adsorption–desorption isotherms of the samples were measured by Tristar II 3020 analyzer (Micromeritics, America). The samples were degassed at 120 °C for 8 h. The pore size distribution and Brunauer–Emmett–Teller (BET) specific surface area (S_BET_) were calculated by the density functional theory (DFT) theoretical model and Equation (1) multi-point BET method.

(1) $$\frac{1}{v\left[\left(\frac{p_{0}}{p}\right)-1\right]}=\frac{c-1}{v_{m}c}\left(\frac{p_{0}}{p}\right)+\frac{1}{v_{m}c}$$

v = absorbed quantity; $p_{0}$ = saturation pressure of absorbate; p = equilibrium pressure of absorbate; c = BET constant = exp$\left(\frac{E_{I}-E_{L}}{RT}\right)$; $E_{I}$ = heat of absorption for the first layer; $E_{L}$ = heat of vaporization.

The thermogravimetric behavior of samples during carbonization was measured by a NETASCH STA449FS synchronous thermal analyzer in helium atmosphere at a heating rate of 10 °C/min coupled with an Agilent QMS403D mass spectrometer (TG-MS). The X-ray photoelectron spectra (XPS) measurements were performed by Thermo Scientific K-Alpha X-ray photoelectron spectroscopy with a scanning monochromatic Al-Ka X-ray source (1486.6 eV), and the binding energy was calibrated by the C1s peak of carbon at 284.8 eV. Fourier transform infrared spectroscopy (FT-IR) was measured by a Perkin Elmer Frontier FTIR spectrometer. The scanning wave number was 400 to 4000 cm^−1^, with an average of 32 scans. The powder X-ray diffraction (XRD) test was carried out on a Rigaku Ultima IV X-ray diffractometer with a scanning rate of 2°·min^−1^ from 10 to 80°.

The thermal diffusion coefficients (α) between 20 and 1900 °C of Φ 10 mm × 2 mm samples were measured using the NETZSCH LFA427 laser flash thermal conductivity apparatus. The thermal conductivity (λ) was calculated by Equation (2), where the specific heat (C_p_) value was consistent with the previous literature [25].

(2) $$\lambda =\alpha \left(Τ\right){\rho C}_{P}\left(T\right)$$

The mechanical properties of the materials were characterized by an MTS C44.104 microcomputer controlled multifunctional experimental machine. The compressive measurement was performed using specimens with a dimension of Φ 12 mm × 15 mm (diameter and thickness) at a compressive rate of 1.0 mm·min^−1^.

## 3. Results and Discussion

### 3.1. Morphology and Structure of Polyimide Aerogels

**Figure 2:**
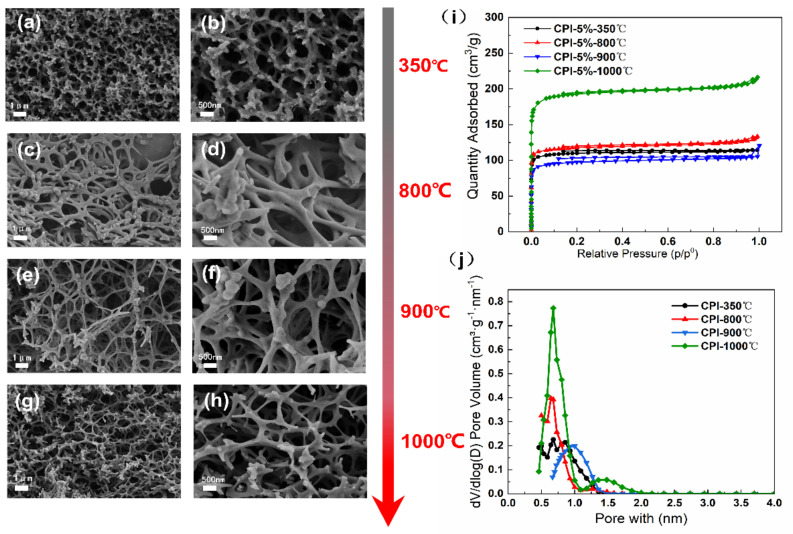
SEM images of the morphology of the aerogels: (**a**,**b**) CPI-350 °C, (**c**,**d**) CPI-800 °C, (**e**,**f**), CPI-900 °C, and (**g**,**h**) CPI-1000 °C; (**i**) N_2_ adsorption–desorption isotherms, (**j**) BJH desorption d*V*/dlog (*D*) pore volume of CPI.

As shown in Figure S1a,b, the dried PI without carbonization was composed of a 3D porous framework with tangled PI aggregates in a pearl chain state (Figure S1a,b). The N_2_ adsorption–desorption isotherm depicted that PI displayed the developed mesoporous and microporous characteristics and has the type-H3 hysteresis loop effects, indicating that PI were typical type IV, as reported by Deng et al. [26] (Figure S1c). The DFT pore size distribution clearly exhibited the presence of mesopores in a PI network (Figure S1d). After being cured at 350 °C for 1 h and further carbonized above 800 °C, the morphology and structure of carbon aerogels were shown in Figure 2. The resultant CPI samples display the fibrous mesh structure with a finer skeleton and smaller pore size in comparison with PI (Figure 2c–h). Additionally, the N_2_ isotherm of the CPI samples are identified as type I [27], proving that there are many micropores in the material (Figure 2i,j).

These changes might be attributed to the weight loss and shrinkage of the aerogel nano-skeleton during the carbonization process, which could give rise to the continuous conversion of mesopores into micropores. It is well acknowledged that the structure of the nanoscale pores plays a vital role in the thermal insulation performance [28]. Thus, we further investigated the pore properties of the samples, as shown in Table 1.

After the aerogel samples went through the carbonization process, the BET specific surface area (S_BET_) significantly increases from 303.589 to 475.105 m^2^/g and subsequently increased to 764.169 m^2^/g with the carbonization temperature further increasing from 800 to 1000 °C. The average pore size (D_pore_) was found to decrease from 13.8 to 4.3 nm and keep reducing to 3.4 nm above 800 °C. As a result, CPI shows a notable rise of micro porosity compared with PI, which kept above 74% under different carbonization temperature conditions.

In particular, the SEM images (Figure 2a,b) and structural properties of CPI (Table 1) show that the aerogel curing at 350 °C for 1 h has a similar specific surface area (446.574 m^2^/g) and pore structure properties as the carbon aerogel carbonized above 800 °C. The constant temperature heat treatment at low temperature has a great influence on the development of the polyimide-based pore structure.

### 3.2. Morphology and Structure Analysis of the Composites

**Figure 3:**
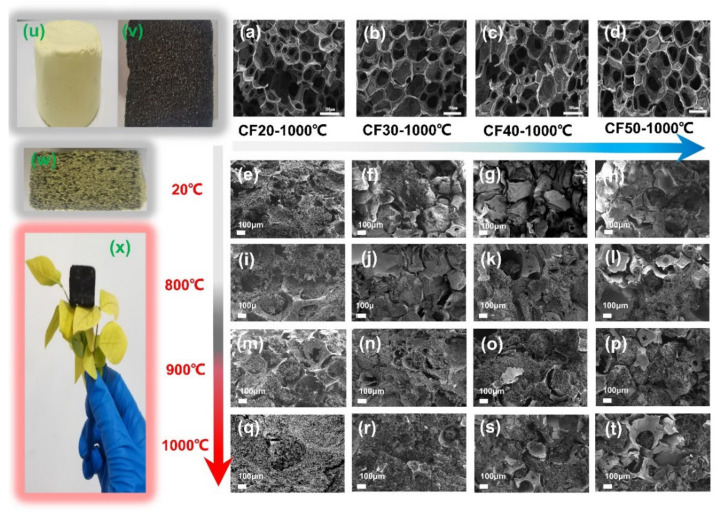
The SEM images of the morphology of carbon foam: (**a**) CF20-1000 °C, (**b**) CF30-1000 °C, (**c**) CF40-1000 °C, (**d**) CF50-1000 °C, and composite: (**e**) CF20-PI, (**f**) CF30-PI, (**g**) CF40-PI, (**h**) CF50−PI, (**i**) CF20-CPI-800 °C, (**j**) CF30-CPI-800 °C, (**k**) CF40-CPI-800 °C, (**l**) CF50-CPI-800 °C, (**m**) CF20-CPI-900 °C, (**n**) CF30-CPI-900 °C, (**o**) CF40-CPI-900 °C, (**p**) CF50-CPI-900 °C, (**q**) CF20-CPI-1000 °C, (**r**) CF30-CPI-1000 °C, (**s**) CF40-CPI-1000 °C, (**t**) CF50-CPI-1000; the photographs of (**u**) PI, (**v**) CF20-500 °C, (**w**) CF20-PI and (**x**) CF20-PI-1000 °C.

It is believed that the type of foam and carbonization temperature can influence the structure of composites such as linear shrinkage and density, which in turn have a significant effect on the thermal conductivity of the composite [29,30,31]. Figure 3 depicts the morphology evolution of carbon foam-reinforced polyimide aerogels composites under different carbonization temperatures. Figure 3w shows that the yellow polyimide aerogel phase is uniformly distributed in the pre-carbonized foam after vacuum impregnation and supercritical drying, which is later transformed into a black carbon aerogel after co-carbonization at 1000 °C (Figure 3x). The micron skeletons of foam reinforcements endow the composites with excellent formability with low density, which mainly achieved the design purpose of the composite materials.

The SEM images show that the composites prepared with different pre-carbonized foam maintain the micron carbon foam skeleton and the nano aerogel-filled structure, but the interfacial gap is different. After co-carbonization, the composites with CF20 and CF30 have smaller interfacial gap than those with CF40 and CF50. Therefore, the interfacial gap size of the composite can be controlled by adjusting the density of the carbon foam.

**Figure 4:**
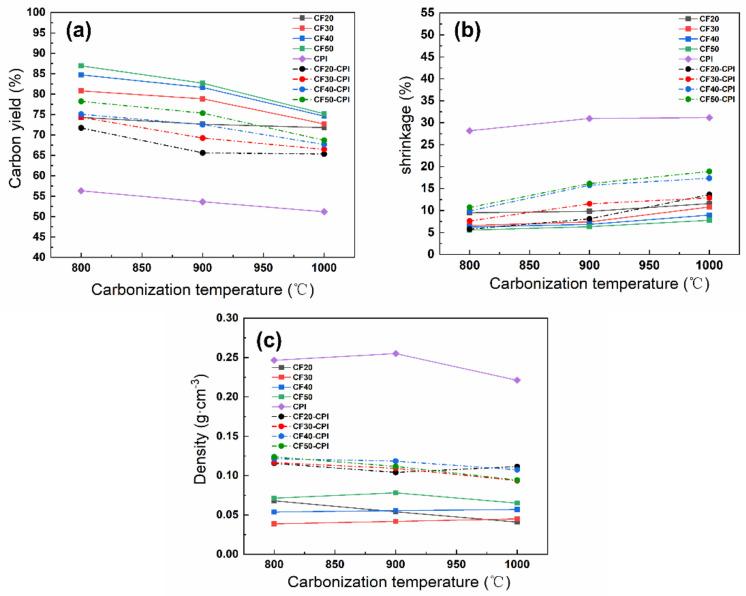
The effect of carbonization temperature on (**a**) the carbon residue rate, (**b**) the shrinkage and (**c**) the density of polyimide aerogels, pre-carbonized foams and composites.

Figure 4a shows that the carbon yield of all the CF-CPI composites decreases with the carbonization temperature rising and approaches to about 70% at 1000 °C, which is similar to the trend of the corresponding CF samples. In addition, the carbon yield of the composites went up with the increment of phenolic resin impregnating solution concentration. It can be observed from Figure 4a that increasing the concentration of impregnating solution of pre-carbonized foam could reduce weight loss of the CF samples and the corresponding CF-CPI composites in the co-carbonization process. CF20 and CF20-CPI possess the lowest carbon yields of carbon foams and composites, while the highest carbon yields of carbon foams and composites belong to CF50 and CF50-CPI. With the raising of carbonization temperature, the carbon yields of CF20 and CF20-CPI decrease from 74% to 71% and 71% to 65%, respectively, which is lower than CF50 and CF50-CPI nearly by 10%. 

The carbonization shrinkage of the carbon foams, aerogels and composites were measured to analyze the evolution of the interfacial gap (Figure 4b). The shrinkage of PI after carbonization was approximately 30% and kept numerically stable above 900 °C. The shrinkage of CF20, CF30, CF40 and CF50 was about 10%, 9%, 7%, and 6%, respectively, indicating that the shrinkage of carbon foams was not significantly affected by carbonization temperature under the experimental conditions but increases with the lower phenolic resin loading. Therefore, the narrower interfacial gap of CF20-CPI (Figure 3i,m,q) and CF30-CPI (Figure 3j,n,r) can be attributed to lower carbon yield and the higher shrinkage of the carbon foam. In addition, the shrinkage of the composite is generally higher than that of the corresponding pre-carbonized foams as the carbonization temperature increased. It was also proved that the carbon foam in the composites was dominated by the high shrinkage and pulling effect of the aerogel matrix phases during the co-carbonization process. These changes lead to a larger co-shrinkage phenomenon of the composites, which could effectively suppress the generation of the interfacial gap.

The density variation of CPI, CF and CF-CPI is shown in Figure 4c. The density of all the composites varies from 0.10 to 0.125 g/cm^3^. Table S1 shows that the density increment of the four composite precursor is similar after being impregnated with polyimide aerogels, indicating that their aerogel mass loading is comparable. After carbonization, a slight decrease in the density of the four composites was observed, which was induced by weight loss and volume shrinkage. The density variation of the composites can be divided into two stages. From 800 to 900 °C, the density of CF, CPI and CF-CPI is largely constant with further increases in the temperature of carbonizing, which indicates that the weight loss of samples is comparable to the ratio of volume shrinkage. From 900 to 1000 °C, since the shrinkage of aerogel-reinforced phase reached numerical stable after 900 °C, the high carbonization temperature only causes the decrease in carbon yield and the decrease in density. The degree of density decline of the composites was generally higher than that of the corresponding pre-carbonized foam. Due to the increased shrinkage of CF20-1000 °C and the small gap between the two phases in the composite sample, the co−shrinkage behavior of CF20-CPI-1000 °C is mainly influenced by the foam. Since the drop of volume exceeded the increasement of weight loss, the density slightly increased with the growth of co-carbonization temperature.

### 3.3. Analysis of the Co-Carbonization Behavior

**Figure 5:**
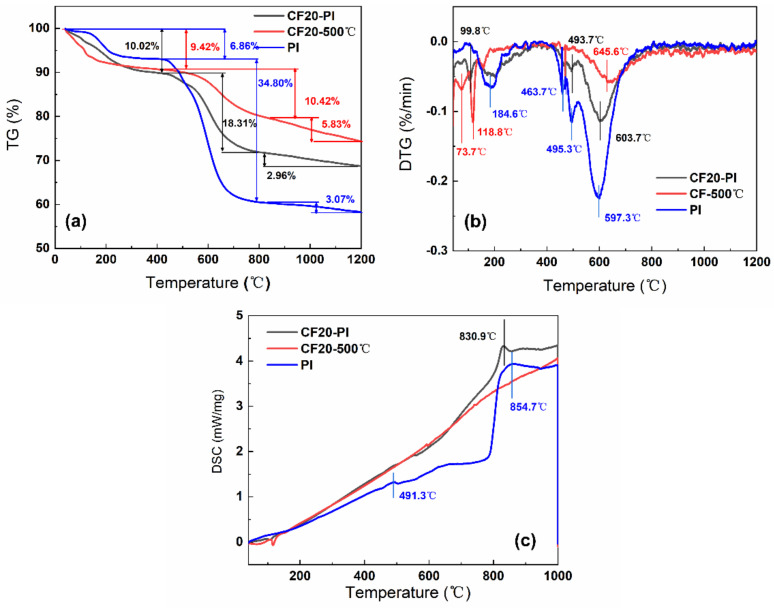
(**a**) Thermogravimetric (TG) curves and (**b**) differential thermal gravity (DTG) curves (**c**) differential scanning calorimetry (DSC) curves of CF20-PI, CF20-500 °C and PI.

**Figure 6:**
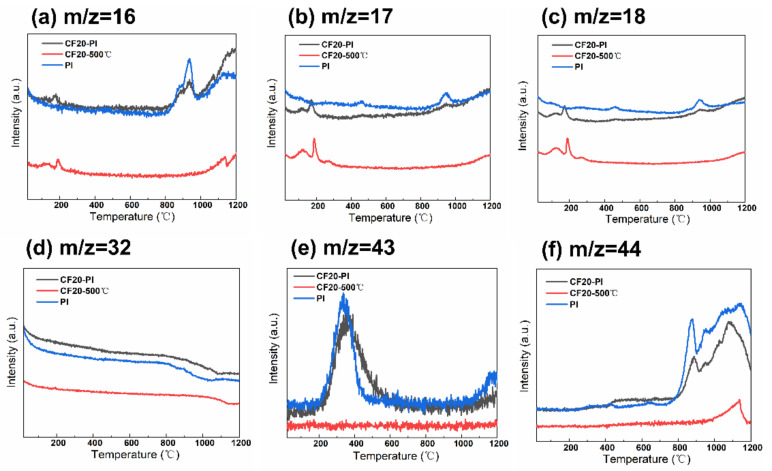
Weight spectrometry images of polyimide aerogels, pre-carbonized foams and composites. (**a**) m/z = 16, (**b**) m/z = 17, (**c**) m/z = 18, (**d**) m/z = 32, (**e**) m/z = 43, (**f**) m/z = 44.

The thermal decomposition behaviors and chemical structure changes during the pyrolysis process of CF20-PI, CF20-500 °C, and PI were investigated by TG-DSC-MS; the results are shown in Figure 5 and Figure 6. Both the PI aerogel phase and the CF20-500 °C pre-carbonized foam phase showed two weight loss stages before carbonization at 800 °C. Above 800 °C, the weight loss became slower, and the pyrolysis reaction was almost complete. For all the samples, the main thermal weight loss occurred around the temperature range from 200 to 700 °C.

The TG curve of CF20-500 °C has two weight loss peaks at 118.8 °C and 645.6 °C, where the weight loss is 9.42% and 10.42%, respectively (Figure 5a). The MS spectral signals of CH_4_ (m/z = 16), NH_3_ (m/z = 17), H_2_O (m/z = 18), and O_2_ (m/z = 32) were detected (Figure 6a–d). This may be related to the decomposition of the residual phenolic macromolecular structure, free phenol groups, as well as the secondary chemical decomposition due to the delayed occurrence of the phenolic resin-coated polyurethane foam backbone—the breakage of the polymeric polyurethane chain structure to produce diisocyanate and polyhydric alcohols, which break down to form amines, olefins and other small molecules [32]. After 800 °C, the weight loss of CF20-500 °C was only about 5%, and there is no significant endothermic peak in the DSC curve of CF20-500 °C. With the increases of temperature, the molecular chain undergoes deoxygenation, and oxygen elements are released in the form of O_2_ (m/z = 32) and CO_2_ (m/z = 44). During the polycondensation and rearrangement of carbon chain backbone, the dangling bonds remaining in the aromatic ring separated from the larger ones to combine with the larger ring structure to form a rigid polycyclic aromatic structure [33]. Thus, the pore size no longer changes significantly during the carbonization at higher temperatures, which is conducive to the retention of the porous structure of the foam [24,32]. The final effective carbon yield of CF20 is about 74.33%, which is similar to the experimentally measured carbon residue rate.

The two pronounced weight loss peaks of PI occur at 184.6 °C and 597.3 °C, with weight loss values of 6.86% and 34.80%, respectively. The first stage of the low weight loss is mainly caused by the desorption of some small molecules such as O_2_ and H_2_O from the aerogel system and the undried solvent. The second stage of weight loss appears only after 450 °C, and the melt endothermic peak of PI appears at 491.3 °C in Figure 5c, which indicates that the thermal stability of PI is good. During this stage, the imide ring undergoes partial ring-opening reaction, the C–N bond, precipitating O=C–NH/O=C–NH_2_ (m/z = 43) with small amounts of NH_3_ (m/z = 17), H_2_O (m/z = 17), which becomes the main nitrogen loss phase with high weight loss [26]. Above 800 °C, the DSC curves of PI shows a significant endothermic peak at 854.7 °C, while the thermal decomposition rate of PI slows down, and only 3.07% weight loss occurs, which indicates that the system undergoes the pyrolysis reaction of deoxygenation and dehydrogenation, which results in the incorporation of aromatic rings [11]. Oxygen is removed mainly in the form of O_2_ (m/z = 32), CO_2_ (m/z = 44). Meanwhile, a small amount of H_2_O (m/z = 18) is generated from ether bond breaking. The effective carbon residue rate of CPI after carbonization is about 55.27%, which is similar to the experimentally measured carbon yield.

The thermal decomposition behaviors of the CF20-PI composite combined with the characteristics of CF20-500 °C and PI go through two thermal weight loss stages successively: the first stage of weight loss temperature range is between 20 and 200 °C, and the weight loss of the composite was 10.02%. In addition, the weight loss temperature range was close to that of the constituent monomer phase. The second stage of weight loss occurred between 425 and 800 °C and the weight loss of this stage reached about 18.31% at 800 °C, which was significantly enhanced comparing with the first stage, and this indicated that there was a chemical reaction resulting in the escape of pyrolysis products. The weight loss of CF20-PI became slower after 800 °C with the endothermic peaks of CF20-PI appearing at 830.9 °C, indicating that the main reactions at this stage are condensation and rearrangement of the carbon chain skeleton. CF20-PI has the final carbon yield of 68.71% at 1200 °C, which is higher than PI and lower than CF20-500 °C, which matches with the experimentally measured value.

In summary, since the shrinkage of the material is related to the weight loss due to chemical reactions [24], the pre-carbonized foam and aerogel have close thermal weight loss temperature ranges and similar types of gaseous cracking products during the carbonization process, which is beneficial to achieve the co-shrinkage of the material during the co-carbonization of the composite to limit the gap size of the two-phases.

### 3.4. Analysis of the Co-Carbonization Products

The X-ray photoelectron spectroscopy (XPS) was employed to identify the surface elemental species and percentage of CF20-CPI, CF20 and CPI at different carbonization temperatures, and the results are shown in Table 2. In general, the content of the three main elements contained in the monomer phase and the composite changed in the same pattern, and the content of carbon was significantly increased while the content of nitrogen and oxygen decreased. This phenomenon becomes more obvious with the rise of carbonization temperature, indicating that the carbonization degree was increasing. Compared with the change of the peak response of IR spectral characterization, the evolution of surface chemical elements corresponds to deoxygenation and hydrogenation during the inorganization of organic polymers. A small amount of nitrogen contained in the products may be present in the nitrogen-containing polyheterocycles observed in the IR characterization [34].

**Figure 7:**
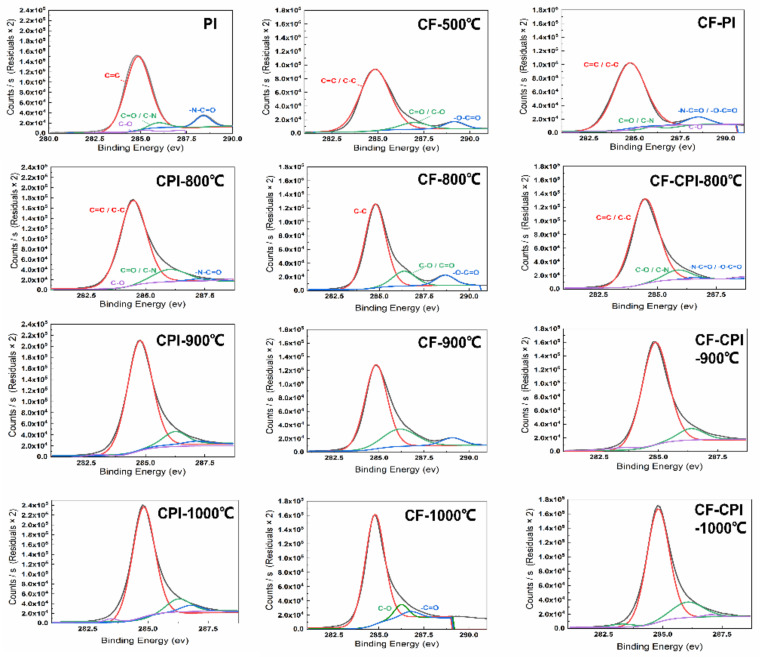
X-ray photoelectron spectroscopy images of polyimide aerogels, pre-carbonized foams and composites.

The results of the high-resolution peaks of C1s in the three types of samples mentioned above are shown in Figure 7. The response peaks of polyimide aerogel are deconvoluted into four individual peaks centered at 284.7 eV (C=C), 285.3 eV (C–O), 286.1 eV (C=O/C–N) and 288.5 eV (–N–C=O) [35]. With the increase in the carbonization temperature, the binding energy of the peak where C=C is located moves to 284.8 eV, and the peak increases. The peaks of –N–C=O and C=O/C–N gradually weaken. The C–O peak maintains at a low level, which may be related to the decomposition of the imide ring, the breaking of the ether bond in the bridging part of the polyimide molecule and the removal of some of the C–C bonds with low binding energy [36,37,38]. The high-resolution peaks of C1s in the pre−carbonized foam can be divided into four chemical positions at 285.0 eV (C=C/C–C), 286.9 eV (C–O/C=O), 289.3 eV (–O–C=O). With the increase in the carbonization temperature, the C=C/C–C response peak at 285.0 eV becomes narrower with an increase in peak width. The –O–C=O content keeps decreasing. The C=O generated by the splitting decomposition of –O–C=O further forms a small amount of C–O. Through the deoxygenation reaction, the content of C–O decreases, which is consistent with the results of the gaseous product test by TG-MS. Such a phenomenon indicates that after carbonization, the methylene bonds remaining in the pre-carbonized foam attached to the benzene ring further undergo dehydration condensation reactions with the hydroxyl. Some of the unreacted methylene bonds are removed in the form of CH_4_. Subsequently, the hydroxyls also dehydrate to form ether bonds, and the hydrogen is released in the form of H_2_O [39].

The composite sample C1s fractionation spectrum combines the characteristics of the two types of monomer phases mentioned above, and the four carbon response peaks of C=C/C–C, C–O, C=O/C–N, and –N–C=O/–O–C=O appear at 284.9, 285.4, 286.0, and 288.6 eV, respectively, for CF20-PI before co-carbonation. In summary, with the increase in the carbonization temperature, the relative content of the C=C bond of the composites increases, and the content of C–O, C=O/C–N and –N–C=O/–O–C=O decreases. It is worth mentioning that the elemental compositions of the products carbonized from CF20, CPI and CF20PI are highly similar, indicating that the co-carbonization process is beneficial to enhance the compatibility of the composites and reduce the interfacial gap between the two phases.

**Figure 8:**
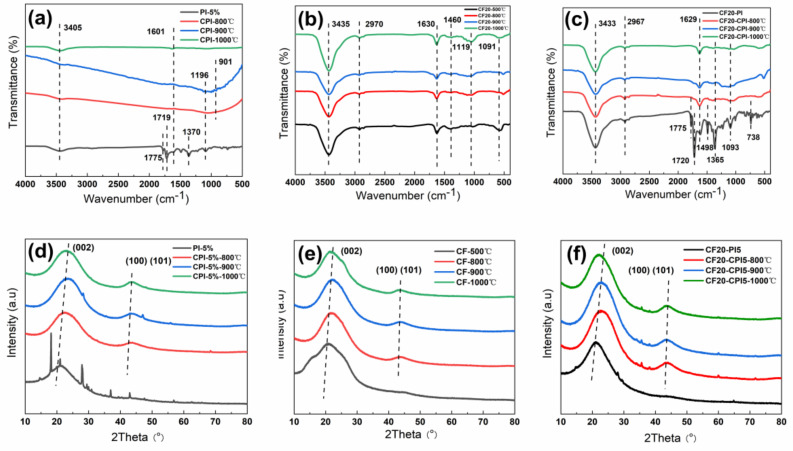
FTIR spectra of (**a**) aerogels, (**b**) foams and (**c**) composites; XRD patterns of (**d**) aerogels, (**e**) foams and (**f**) composites.

To analyze the chemical bonds of the carbonization products of polyimide aerogel, pre-carbonized foam and carbon foam-reinforced polyimide-based carbon aerogel composite carbonized at different temperatures, the three types of samples were characterized by infrared spectroscopy, and the results are shown in Figure 8a−b.

It can be seen in Figure 8a that the characteristic peaks of the imide rings at 1775, 1720, 1370, and 721 cm^−1^ disappear in the polyimide-based carbon aerogels obtained after carbonization. The response intensity of the C=C bond quadrant stretching peak in the benzene ring at 1601 cm^−1^ weakens, which implies the cleavage and escape of various functional groups, including imide structures. The honeycomb carbon chain structure formed by recombination increases [11]. Meanwhile, the stretching vibration peak of the N-H appears at 3405 cm^−1^, indicating that trace amounts of N elements were still present within the carbonized sample. A wide peak between 1196 and 901 cm^−1^ at 800 and 900 °C may be associated with the periodic stretching vibration of the C–O–C bond and the out-of-plane bending vibration of the C–H bond in the sp^2^ hybrid orbital [35,40]. With the increase in the carbonization temperature, the deoxygenation rate and unsaturated bond breakage of the aerogels increased, which is consistent with the results of TG-MS and XPS characterization. According to existing reports, the thermal decomposition reaction occurs while the structure of dense ring aromatics was gradually generated through thermal polycondensation reaction and transformed to a planar mesh structure to form an amorphous carbon structure [26,41]. In addition, the signal of each spectral peak becomes weaker when raising the carbonization temperature, which also proves that the carbon aerogel has high extinction characteristics in the infrared light region.

As can be seen from Figure 8b, the infrared spectral curves of the pre-carbonized foam pyrolysis products at each temperature have the same peak positions and shapes. There are obvious absorption peaks of hydroxyl stretching and bending vibrations at 3435 and 1630 cm^−^^1^. The C–H bond and C–O–C methylene ether bond stretching vibrational response in the methylene are located at 2976, 1460, and 1119 cm^−1^. The characteristic stretching vibration peaks of the C–C bond and the C–O bond of the phenolic hydroxyl group in the benzene ring at 1610 and 1210 cm^−1^ wave numbers weaken with the increases of carbonization temperature, which indicates that the benzene ring structure and the methylene ether and methylene bonds in the phenolic resin skeleton network have been broken. The products are converted into inorganic compounds with high carbon content [42].

As can be seen from Figure 8c, the chemical bonds of the composite samples at different carbonization temperatures combine the main features of the corresponding aerogel and carbon foam phases. For CF20-PI, the peaks located at 1775, 1720, 738, and 1365 cm^−1^ can be assigned to the he asymmetric stretching, symmetric stretching, bending vibrations and the stretching vibration of C–N–C of imide carbonyl groups, respectively [11]. The absorption peaks of the hydroxyl stretching and bending vibrations belonging to the pre-carbonized foam reinforced phase occur at 3433 and 1629 cm^−1^.

After carbonization, the peak positions and peak shapes of the spectra are basically consistent with those of the pure carbon foam, but the intensity of peaks is weakened due to the enhanced infrared shielding effect of the carbon aerogel enhanced phase. Compared to pure carbon foams, the composites have increased infrared extinction coefficient of material by combining the two phases, which can reduce the radiative thermal conductivity of the composite material under high-temperature conditions and obtain carbon foam-reinforced polyimide-based carbon aerogel composite with excellent high temperature thermal insulation properties. In addition, compared with the spectrum of pure carbon aerogel in Figure 8a, the C–N–C stretching vibration peak of the aerogel phase at 1365 cm^−1^ in the composite after carbonization is preserved, indicating that there are some nitrogen−containing polyhedral in the composite sample.

XRD was performed to study the surface crystallographic changes of the three types of samples, and the results are shown in Figure 8d–f. As shown in Figure 8d, compared with the polyimide aerogel, the three characteristic peaks of the crystalline surface diffraction peaks at 15.5°, 22.5°, and 25.5°, which correspond to (10), (010), and (117), respectively, disappeared in carbon aerogel after carbonization above 800 °C.

In Figure 8e, the carbon foams appear to have (002) amorphous carbon crystalline surface diffraction peaks and (101) graphitic structure peaks with short and fat peaks in the intervals of 20.5° to 23.5°, 42.5° to 43.5° [24]. Moreover, with an increase in the carbonization temperature, the (002) crystalline diffraction peaks experience the red shift, the (101) crystalline diffraction peaks become narrow and the intensity of the peaks increases. These may be related to the partial destruction of the amorphous carbon structure, the formation of highly oriented hexagonal carbon, and the tendency of the material to increase its crystallinity and develop toward graphitization [11,36]. In addition, the diffraction curve of the carbon foam in Figure 8e tends to smooth out with only the two carbon structure diffraction peaks mentioned above and the intensity of its response signals increases, which indicates that high temperature heat treatment above 800 °C can effectively remove the heteroatoms except for the carbon atoms from the pre-carbonized foam body to form a reticular glassy carbon foam dominated by amorphous carbon [24].

Figure 8f shows the XRD patterns of the composite sample of carbon foam-reinforced polyimide-based aerogel composites at different carbonization temperatures. The XRD patterns of the composites demonstrate that the aerogel remains amorphous even after pyrolysis at 900 °C because of the broad diffuse reflection peaks near 24° and 44° relative to the (002) and (100) (101) diffraction planes of the graphite layers, respectively. With the raising of carbonization temperature, the variation of the characteristic peaks of the composites were consistent with the change pattern of the two-phase monomer, which showed a trend of narrowing and red shift. These imply a crystalline structure dominated by amorphous carbon.

### 3.5. Heat-Shielding Performance

**Figure 9:**
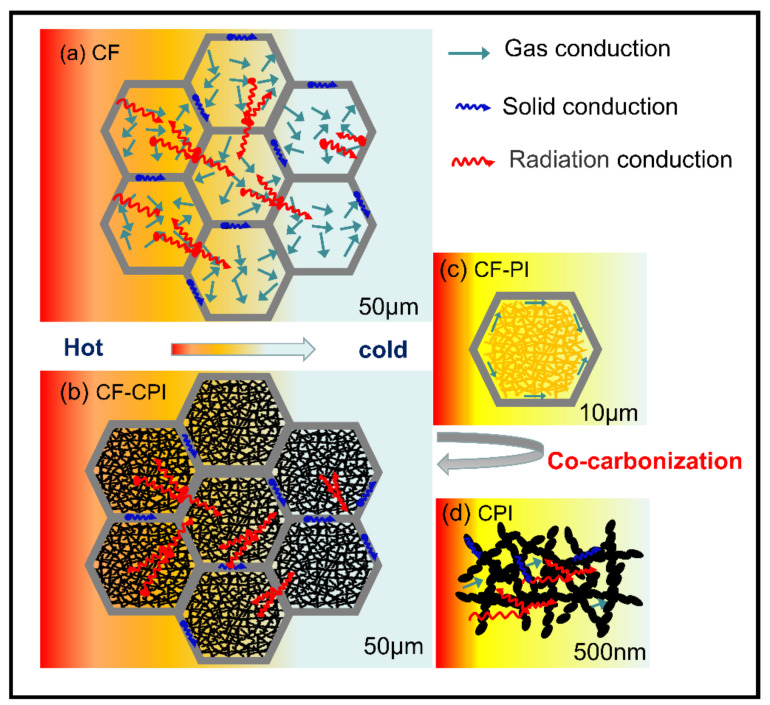
Schematic illustration of heat transfer mechanism (**a**) CF, (**b**) CF-CPI, (**c**) CF-PI, (**d**) CPI.

Figure 9 describes the heat transfer mechanisms of the highly porous monolithic materials including gaseous thermal conduction, solid state thermal conduction and radiative thermal conduction. The level of thermal conductivity is closely related to the skeleton density, extinction coefficient, and pore structure of the porous material [4,43,44,45]. In the previous study, the polyimide-based carbon aerogel has a higher microporosity than that of conventional phenolic resin-based carbon aerogel observed in Table S2 [16], which affects the thermal conductivity of the materials through three pathways: scattering of phonons, blocking of photons, and inhibition of gas molecule collisions by nano-skeleton particles as well as nanopore size structure. In this work, the thermal diffusivity of samples prepared at different carbonization temperatures and different pre-carbonized foams were measured under an argon atmosphere within the temperature range of 25–1900 °C (Table S3), and thermal conductivity was calculated according to Equation (2), using measured density and the specific heat reported elsewhere [25]. Thermal conductivity–temperature curves (Figure 9) show that the effect of carbon aerogel on the thermal insulation enhancement of composite samples can be divided into two stages.

**Figure 10:**
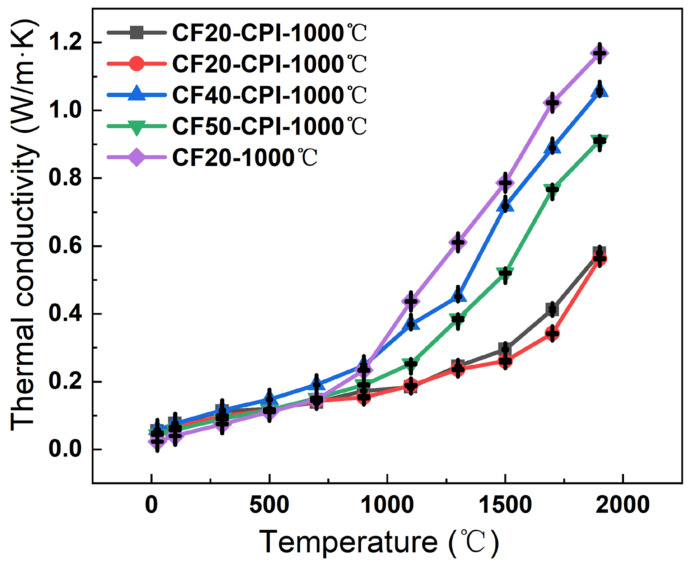
Thermal conductivity of CF and CF-CPI samples with the test temperature from 25 to 1900 °C.

Below 900 °C, the thermal conductivity is mainly influenced by the thermal conductivity of solid and gaseous states, which is related to the density and pore structure of the material (Figure 10) [46]. At lower temperatures, the thermal conductivity of the composites was close to that of the carbon foams. This may be the result of the facilitation of solid-state heat transfer caused by the increased density of the material offset by the inhibition of gaseous heat transfer due to the nanopore size of the aerogel being lower than the free range of air molecules, which limits the heat transfer by collision of gas molecules. In addition, the four composites have similar thermal conductivity at this stage, ranging from 0.05 to 0.12, which increased slightly with the raising of test temperature.

Above 900 °C, the high-temperature radiative thermal conductivity gradually dominates the thermal conductivity as the temperature increases. The nanostructure of the carbon aerogel is smaller than the average free range of photons, which makes the photons that originally propagate along the pore wall of the carbon foam continuously absorbed and re-radiated by the carbon particles of the solid skeleton in all directions during the propagation process, achieving smaller energy transfer [7]. As a result, the composites have a higher specific extinction coefficient, and their thermal conductivity above 900 °C is significantly lower than that of the carbon foams. The measured thermal conductivity of the four composites carbonized at 1000 °C numbered in the order: CF30-CPI-1000 °C < CF20-CPI-1000 °C < CF50-CPI-1000 °C < CF40-CPI-1000 °C. CF20-CPI-1000 °C and CF30-CPI-1000 °C exhibit high-temperature thermal conductivity of 0.57 and 0.56 W/m⋅K at 1900 °C, respectively. Compared with CF40-CPI-1000 °C (1.06 W/m⋅K) and CF50-CPI-1000 °C (0.91 W/m⋅K), the thermal conductivity of the composites prepared by CF20 and CF30 reduced by nearly 40%. Combining with the density change and the microstructure of the material (Table S1, Figure 3), this may be related to the inhibition of the low-density pre-carbonized foam on the propagation of phonons across the pore walls and the restrictive effect of the smaller interfacial gap on the collisional heat transfer between gas molecules, indicating that low-density foam reinforcement is beneficial to improve the high-temperature thermal insulation performance of the composite. In addition, compared with the similar as-prepared insulated materials (Table 3), the phenolic resin-based carbon foam enhanced polyimide-based carbon aerogel has an excellent high-temperature insulation human performance.

### 3.6. Mechanical Properties

The mechanical property of insulation materials is an important index for its practical applications. Here, the compression tests were carried out on CF-CPI-1000 °C samples and the corresponding CF matrix to study the mechanical reinforcement effect of the aerogel on the conforming material after co-carburization and the effect of the pre-carbonized matrix foam type on the mechanical properties of the composite samples.

**Figure 11:**
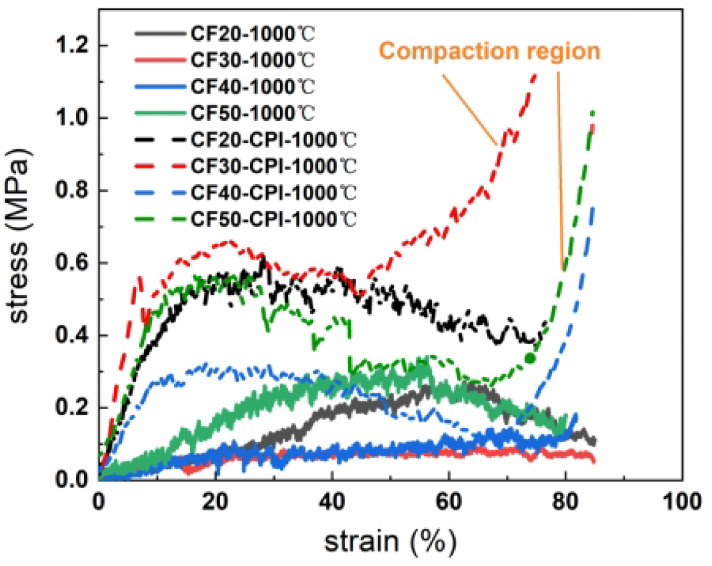
Compressive strain–stress curves of CF-1000 °C and CF-CPI-1000 °C.

Figure 11 shows the stress–strain curves of CF-CPI-1000 °C and CF-1000 °C. The compressive behavior of the composite-reinforced structure formed by filling the carbon aerogel-reinforced phase with a 3D network composed of crosslinked nanoparticles into the hexagonal glassy pore of carbon foam is similar to that of traditional polymers [49]. The stress–strain curve of CF-CPI can be divided into three stages: (1) The composite appears in the elastic deformation area at the low strain stage [50]. (2) When the strain approaches 10%, the composite enters the yield plateau area, which is the main stage of energy absorption of materials. (3) When the strain reaches about 70%, the stress–strain curve approaches the densification failure point, and the fracture mode of the composite becomes plastic. In addition, there is no compaction region in the stress–strain curve of CF, indicating that the brittle failure of CF happens at the end of plateau area due to a sudden fracture.

**Figure 12:**
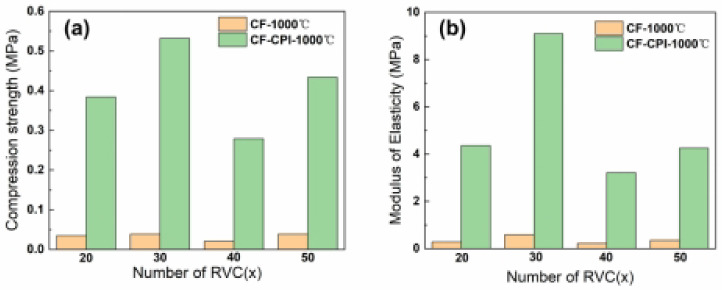
Compressive strength (**a**) and elastic modulus (**b**) of CF and CF-CPI-1000 °C.

The compressive strength and elastic modulus of CF-CPI-1000 °C and the corresponding CF-1000 °C are shown in Figure 12. Comparing with the pure carbon foam, the compressive strength and elastic modulus of all composites are significantly improved by the incorporation of the highly cross-linked aerogel phase. However, the mechanical properties of the prepared composites are not adequate for single structural application, which may be more suitable for the interlayers of sandwiched insulations. As shown in Figure 12b, the ultimate compressive strengths of CF30-CPI-1000 °C and CF50-CPI-1000 °C are 0.532 MPa and 0.434 MPa, while the ultimate compressive strengths of CF20-CPI-1000 °C and CF40-CPI-1000 °C are 0.384 MPa and 0.279 MPa. The ultimate elastic modulus of CF30-CPI-1000 °C and CF50-CPI-1000 °C are 9.091 MPa and 4.251 MPa, while the ultimate elastic modulus of CF20-CPI-1000 °C and CF40-CPI-1000 °C are 4.316 MPa and 4.251 MPa.

## 4. Conclusions

In this work, polyimide aerogels with nanoscale porous structures were impregnated into the micron-scale pore of pre-carbonized carbon foam, and carbon foam-reinforced polyimide-based carbon aerogel composites were prepared by co-carbonization under different carbonization temperatures. The polyimide aerogel cured at 350 °C forms a stable microporous structure, which effectively inhibits the collapse of the nano-skeleton of the aerogel during the subsequent carbonization process above 800 °C. Despite the carbonization shrinkage mismatch between pure polyimide aerogel and pure phenolic resin-based pre-carbonized foam, the interaction forces between the two phases of the composite sample effectively inhibit the interfacial separation. Under the conditions of carbonization process, the interfacial gap between CF20-CPI and CF30-CPI was low and reached a minimum value at 1000 °C due to the low carbon yield and the high shrinkage of the foam. Raising the carbonization temperature and reducing the density of pre-carbonized foam are beneficial to increase the shrinkage of the pre-carbonized foam, which in turn inhibits the development of the gap between the two phases in the composite samples. In addition, the main weight loss of the aerogel, pre-carbonized foam and composite samples occur in similar temperature ranges, and the functional groups and crystalline distribution of solid products of the composite samples during the carbonization process conformed well to the characteristics of the two phases. The final composites were found to be more compatible with each other, which is beneficial to inhibit the separation of phase interfaces in the composite sample. Among the composites, CF30-CPI-1000 °C showed the best thermal insulation performance under high temperature, with a low thermal conductivity of 0.56 W·(m·K^−1^) at 1900 °C. Meanwhile, CF30-CPI-1000 °C showed a remarkable increase in both of compressive strength and elastic modulus (0.532 MPa, 9.091 MPa respectively) in comparison with the pure foam matrix (0.039 MPa, 0.59 MPa respectively). Considering the excellent performance, the composite should have potential to be used as a structural–functional integrated carbon material for aerospace thermal protection with low thermal conductivity, excellent moldability and light weight.

## Figures and Tables

**Table 1:** Structural properties of PI and CPI.

Sample	S_BET_ (m^2^/g)	S_mes_ (m^2^/g)	S_mic_ (m^2^/g)	V_total_ (cm^3^/g)	V_mic_ (cm^3^/g)	D_pore_ (nm)	Microporosity (%)
PI	303.589	294.167	9.422	1.301	0.003	13.8	0.21
CPI-350 °C	446.574	55.945	390.628	0.177	0.146	3.4	82.49
CPI-800 °C	475.105	52.660	422.445	0.206	0.159	4.3	77.18
CPI-900 °C	391.075	66.247	324.826	0.163	0.122	3.6	74.85
CPI-1000 °C	764.169	97.693	666.475	0.335	0.256	3.4	76.42

**Table 2:** Quantitative analysis of the surface C, O and N atomic percentages of polyimide aerogels, pre-carbonized foams and composite samples and their carbides under different carbonization temperatures.

Sample	Atom%		
	C1_S_	O1_S_	N1_S_
PI	81.53	12.71	5.76
CPI-800 °C	91.28	5.45	2.47
CPI-900 °C	92.72	4.89	1.44
CPI-1000 °C	94.17	4.45	0.98
CF20-500 °C	77.94	16.41	3.01
CF20-800 °C	79.78	16.56	2.07
CF20-900 °C	81.47	13.79	3.21
CF20-1000 °C	89.01	7.66	2.17
CF20-PI-500 °C	80.42	15.65	3.93
CF20-CPI-5%-800 °C	87.06	9.57	3.35
CF20-CPI-5%-900 °C	91.46	4.81	3.22
CF20-CPI-5%-1000 °C	93.04	4.48	1.57

**Table 3:** Thermal conductivity of as-prepared insulated materials.

Carbon Aerogel Precursor	Carbon Foam Precursor	Thermal Conductivity (W·m^−1^·K^−1^)	Density (g·cm^−3^)	Reference
Phenolic resin	Polyurethane	0.1 (550 °C)	0.05–0.35	[47]
Phenolic resin	Phenolic resin	0.6 (1593 °C) 0.8 (1950 °C)	0.07	[48]
Polyimide	Phenolic resin	0.56 (1900 °C)	0.094	This paper

## Supplementary Materials

The following supporting information can be downloaded at: https://www.mdpi.com/article/10.3390/gels8050308/s1. Figure S1. (a,b) SEM images of the morphology of PI,(c) N2 adsorption-desorption isotherms, (d) BJH desorption dV/dlog(d) pore volume of CPI. Table S1. The density of polyimide aerogels, pre-carbonized foams and composite samples before and after carbonization. Table S2. Textual properties of phenolic resin-based carbon previous research. Table S3. Specific heat and thermal diffusion coefficient of CF-CPI and CF at different temperatures.
