# Supplementary material for: Carbon Foam-Reinforced Polyimide-Based Carbon Aerogel Composites Prepared via Co-Carbonization as Insulation Material
Source: Gels. 2022 May 16;8(5):308. doi: 10.3390/gels8050308 (PMC9141059; doi:10.3390/gels8050308)
Supplement: Supplementary file 1 [file gels-08-00308-s001.zip › gels-1697313-supplementary.pdf]

gels

MDPI

Article

Carbon Foam-Reinforced Polyimide-Based Carbon Aerogel Composites Prepared via Co-Carbonization as Insulation Material

Zixuan Zheng, Guojie Liang *, Li Li, Jing Liu, Xinbo Wang, Yi Sun and Kai Li *

State Key Laboratory of NBC Protection for Civilian, Research Institute of Chemical Defense, 35 Huayuan North Road, Haidian District, 100083 Beijing, China; zzx19972022@163.com (Z.Z.); lily97@buaa.edu.cn (L.L.); liuj200721296@sina.com (J.L.); wxb1993@mail.ustc.edu.cn (X.W.); 13501260901@139.com (Y.S.)

* Correspondence: liangguojie@126.com (G.L.); 750455@sohu.com (K.L.)

Supplementary Material

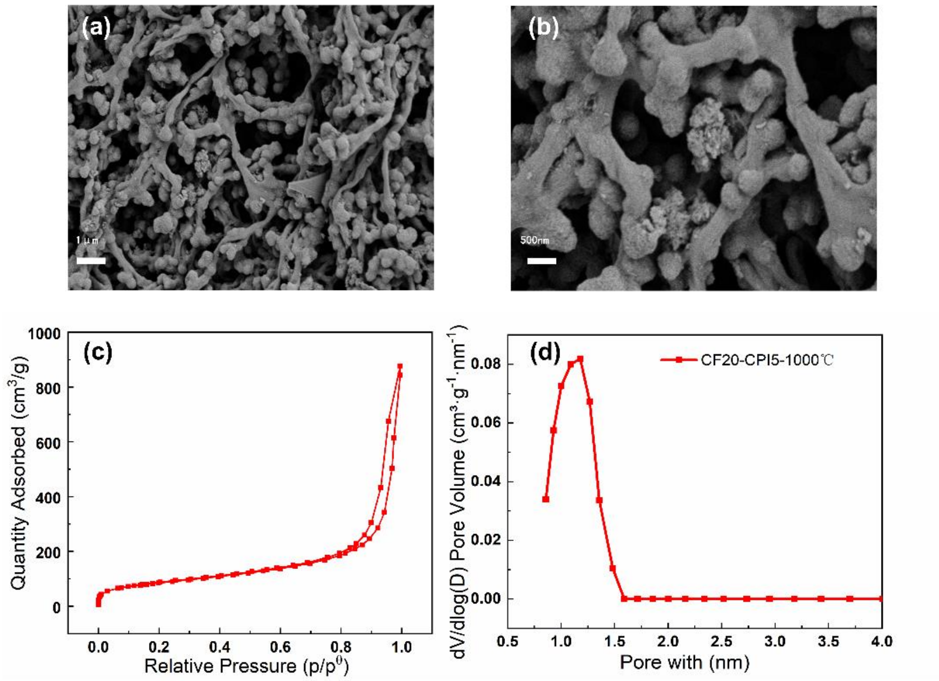

Figure S1. (**a**,**b**) SEM images of the morphology of PI,(**c**) N2 adsorption-desorption isotherms, (d) BJH desorption dV/dlog(**d**) pore volume of CPI.

Table S1. The density of polyimide aerogels, pre-carbonized foams and composite samples before and after carbonization.

Sample	$\rho_{\text{before carbonization}}$ (g·cm^{-3})	$\rho_{800°C}$ (g·cm^{-3})	$\rho_{900°C}$ (g·cm^{-3})	$\rho_{1000°C}$ (g·cm^{-3})
CF20-PI	0.118	0.115	0.105	0.112
CF30-PI	0.124	0.116	0.110	0.094
CF40-PI	0.129	0.121	0.118	0.107
CF50-PI	0.151	0.124	0.117	0.094
CF20	0.037	0.068	0.054	0.041
CF30	0.042	0.039	0.042	0.045

CF40	0.045	0.054	0.056	0.057
CF50	0.071	0.071	0.078	0.065
PI	0.186	0.247	0.255	0.221

Table S2. Textual properties of phenolic resin-based carbon previous research.

**Sample**	S_{BET} **(m²/g)**	V_{total}**(cm³/g)**	V_{mic}**(cm³/g)**	D_{pore} **(nm)**	**Microporosity(%)**	**Reference**
A-900	611.7	0.72	0.25	4.73	34.6	33

Table S3. Specific heat and thermal diffusion coefficient of CF-CPI and CF at different temperatures.

**Temp. /℃**	C_P **(J·(g·K)⁻¹)**	$\alpha_{CF20\text{-}CPI\text{-}1000℃}$ **(mm²·K⁻¹)**	$\alpha_{CF30\text{-}CPI\text{-}1000℃}$ **(mm²·K⁻¹)**	$\alpha_{CF40\text{-}CPI\text{-}1000℃}$ **(mm²·K⁻¹)**	$\alpha_{CF50\text{-}CPI\text{-}1000℃}$ **(mm²·K⁻¹)**	$\alpha_{CF20\text{-}1000℃}$ **(mm²·K⁻¹)**
25	0.71	0.69±0.01	0.74±0.02	0.76±0.01	0.65±0.01	0.8±0.01
100	0.91	0.75±0.02	0.78±0.01	0.78±0.02	0.67±0.01	1.08±0.01
300	1.33	0.76±0.01	0.79±0.02	0.81±0.01	0.72±0.01	1.37±0.02
500	1.56	0.69±0.01	0.8±0.01	0.88±0.01	0.79±0.02	1.73±0.01
700	1.76	0.71±0.02	0.87±0.01	1.01±0.01	0.91±0.01	2.03±0.01
900	1.87	0.83±0.01	0.88±0.02	1.23±0.02	1.08±0.01	3.05±0.02
1100	1.94	0.85±0.02	1.04±0.01	1.77±0.01	1.38±0.01	5.49±0.01
1300	2.00	1.1±0.01	1.26±0.01	2.1±0.01	2.04±0.02	7.45±0.01
1500	2.05	1.29±0.01	1.36±0.02	3.26±0.01	2.69±0.01	9.36±0.01
1700	2.09	1.77±0.02	1.75±0.01	4.66±0.01	3.89±0.01	11.94±0.02
1900	2.11	2.46±0.01	2.85±0.01	4.96±0.02	4.58±0.02	13.51±0.01
